# Allele and genotype frequencies of the *SOD1* gene polymorphism associated with canine degenerative myelopathy in Belgian Malinois dogs in Greece

**DOI:** 10.14202/vetworld.2021.1472-1479

**Published:** 2021-06-09

**Authors:** Antonia Mataragka, John Ikonomopoulos, Georgios S. Zervas, Christos D. Vamvakidis, Nikolaos Tzimotoudis, Ariadne Loukia Hager-Theodorides, Maria Gazouli, Antonios Kominakis

**Affiliations:** 1 School of Animal Biosciences, Department of Animal Science, Laboratory of Anatomy and Physiology of Farm Animals, Agricultural University of Athens, Athens, Greece; 2 Hellenic Army Biological Research Center/Veterinary Clinic, Athens, Greece; 3 Hellenic Army Biological Research Center/Laboratory of Microbiology, Athens, Greece; 4 School of Animal Biosciences, Department of Animal Science, Laboratory of Animal Breeding and Husbandry, Agricultural University of Athens, Athens, Greece; 5 School of Medicine, Department of Basic Medical Science, Laboratory of Biology, Kapodistrian University of Athens, Athens, Greece

**Keywords:** degenerative myelopathy, dogs, genetic analysis, genetic polymorphism, restriction fragment length polymorphism-polymerase chain reaction, *SOD1:c.118A*

## Abstract

**Background and Aim:**

Canine degenerative myelopathy (CDM) is an adult-onset fatal disorder associated with a point mutation of the superoxide dismutase 1 (*SOD1*) gene (*SOD1:c.118G>A*). This study aimed to determine the allele and genotype frequencies of this mutation in a group of Belgian Malinois dogs in Greece.

**Materials and Methods:**

Samples (n=72) of whole blood were collected from 72 purebred dogs of the Hellenic Armed Forces; these samples were processed for DNA isolation, polymerase chain reaction, and digestion with the restriction endonuclease *AcuI*. Sample testing was conducted in compliance with ISO17025 accreditation requirements.

**Results:**

The observed relative genotype frequencies were 71% for the homozygous (GG), 25% for the heterozygous (AG), and 4% for the homozygous mutant (AA) alleles. These frequencies were close to those expected, indicating no significant departure from Hardy–Weinberg equilibrium (HWE, p=0.395). The frequency of heterozygous animals indicates that a high risk of developing CDM in forthcoming generations exists in the tested population because mating among carriers would result in 25% AA progeny. The medical record of the group of study animals indicated selection against leishmaniosis, as applied throughout generations by owners and breeders. The potential association of this selection with the HWE status of the study population was discussed.

**Conclusion:**

The *SOD1:c.118G>A* mutation was common in the tested group of dogs; thus, they are suitable for a follow-up investigation on the development and progression of CDM. A case-control study on animals with evidence of sensitivity to infectious myelopathy could provide new insights into disease pathogenesis.

## Introduction

Canine degenerative myelopathy (CDM) is an adult-onset fatal neurodegenerative disorder characterized by progressive motor neuron loss and paralysis [1,2]. The clinical onset of the disease usually occurs in affected individuals aged >8 years, but definitive diagnosis is only possible postmortem [3]. CDM was initially described in German Shepherd dogs [1,4,5] but has since been diagnosed in several other breeds of dog, including Pembroke Welsh Corgi, Boxer, Chesapeake Bay Retriever, and Rhodesian Ridgeback [2,6-9].

CDM has been associated with a point mutation of the canine superoxide dismutase 1 (*SOD1*) gene (*SOD1:c.118G>A*) [2,7], which shows an autosomal recessive inheritance pattern with incomplete (reduced) penetrance [2]. Animals homozygous for the mutant allele (AA) have a strong predisposition for developing CDM [10], whereas carriers of the mutant allele, that is, heterozygotes (AG), are at low risk, probably because the disorder develops too slowly to become clinically apparent within the usual life span of a dog [2].

In humans, *SOD1* variants have been implicated in the pathogenesis of familial amyotrophic lateral sclerosis (ALS), a debilitating neurological disorder characterized by progressive degeneration of motor neurons; this disease is considered the human analog of CDM [11-13]. *SOD1* is a cytosolic and mitochondrial antioxidant enzyme that protects cells from the toxicity of reactive oxygen species [14]. Mutant *SOD1* tends to form intracellular aggregates due to misfolding, inducing accumulation in the affected motor neurons of another intracellular protein, namely, disulfide isomerase [15].

In the present study, we investigated the genetic basis of CDM in a dog breed, Belgian Malinois, with an increased predisposition to this disease, and a group of animals maintained under a strict program of health surveillance. The information provided was assessed with the goal of identifying risk factors potentially associated with CDM and ALS. This study aimed to determine the allele and genotype frequencies of the *SOD1:c.118G>A* mutation in a group of Belgian Malinois and to compare the results with those previously recorded in relation to the same breed and other breeds.

## Materials and Methods

### Ethical approval

The biological material that was submitted to this investigation was collected exclusively for the purpose of the routine health monitoring of the animal subjects (service dogs of the Hellenic Armed Forces) and not for the purpose of this study. The latter did not involve handling of live of dead animals. According to the applicable legislation (Research Ethics Committee of the Agricultural University of Athens), ethics approval was not required for this study.

### Study period and location

The study was conducted from July to October 2018. Samples (n=72) of whole blood from 72 purebred Belgian Malinois dogs (one sample of 3–6 ml per animal) of the Hellenic Armed Forces were collected from Athens, Greece. The samples were processed at the Laboratory of Anatomy and Physiology of Farm Animals of the Agricultural University of Athens, Greece.

### Sample collection

All animals were 2-7 years of age and healthy, based on a yearly assessment consisting of a clinical examination, biochemical blood analysis, and radiography of the thorax and limbs (which is primarily focused on assessing hip dysplasia). Blood samples were taken from the jugular vein and immediately divided into two portions, one of which was stored at 4-6°C for a maximum of 48 h until it was submitted for DNA isolation. The other portion was stored at −20°C and used as stock.

### DNA isolation

Blood samples were processed for DNA isolation using a commercially available kit and according to the manufacturer’s instructions (NucleoSpin^®^ Tissue, Macherey-Nagel GmbH and Co. KG, Germany). Isolated DNA was stored at −20°C and then used for polymerase chain reaction (PCR). The quality of the isolated DNA was assessed in terms of its purity and integrity through agarose gel electrophoresis, followed by image analysis using a Bio-Rad ChemiDoc XRS+ Molecular Imager (Bio-Rad Laboratories Inc., USA), whereas spectrophotometry was used to measure optical density at 260/280 nm through a NanoDrop 8000 Spectrophotometer (Thermo Fisher Scientific Inc., USA). Fragmented DNA products were discarded, and DNA isolation was repeated from stock samples. DNA isolation and PCR were conducted in compliance with ISO17025 accreditation requirements.

### PCR-restriction fragment length polymorphism (RFLP) analysis

PCR for the amplification of a 296-base pair (bp) fragment from the *SOD1* gene containing the targeted region (*SOD1:c.118G>A*) was conducted according to the previous methods [8] using the Invitrogen Taq DNA Polymerase Kit protocol (Thermo Fisher Scientific Inc.). The reaction mixture consisted of 1×PCR buffer, 0.75-U Taq DNA polymerase, 0.2 mM dNTPs, 1.5 mM MgCl_2_, 0.5 mM of each of two primers (forward: 5′-AGTGGGCCTGTTGTGGTATC-3′; reverse: 5′-TCTTCCCTTTCCTTTCCACA-3′), 5 μl of DNA template, and PCR-grade water to a final volume of 50 μl. The thermal profile of the reaction was that proposed by Holder *et al*. [8]. PCR was conducted using an Applied Biosystems Verity 96-Well Thermal Cycler (Thermo Fisher Scientific Inc.).

PCR products were incubated at 37°C for 1 h with the restriction endonuclease *AcuI* (New England Biolabs Inc., USA). The digestion products were submitted to agarose gel electrophoresis with a 100-bp molecular weight ladder (Nippon Genetics, Europe GmbH) using 3% ultrapure agarose gel (Thermo Fisher Scientific Inc.) stained with ethidium bromide (0.5 μg/mL). The results were assessed using a Bio-Rad ChemiDoc XRS+ Molecular Imager (Bio-Rad Laboratories Inc.).

For specificity confirmation, approximately 20% of the PCR-RFLP products were submitted for sequence analysis, which was conducted on both strands using the Applied Biosystems BigDye Terminator Cycle Sequencing Kit and a PRISM 377 DNA Sequencer (Thermo Fisher Scientific Inc.). The results were compared against deposited sequences in the GenBank database using Basic Local Alignment Search Tool from the National Center for Biotechnology Information.

### Genetic analysis

Allele and genotype frequencies were estimated using the Genepop (online ver. 4.7) software (Michel Raymond and Francois Rousset, Laboratiore de Genetique et Environment, Montpellier, France) [16], which was also used to conduct an exact probability test for Hardy–Weinberg equilibrium (HWE) using the Markov chain method. The assessment of the F_IS_ fixation index was conducted as previously described [17]. F_IS_ is a measure of the average departure of the observed from the expected (under the assumption of HWE) frequencies with theoretical values ranging from −1.0 (all individuals heterozygous or entirely outbred) to +1.0 (no observed heterozygotes or entirely inbred).

## Results

**Figure-1:**
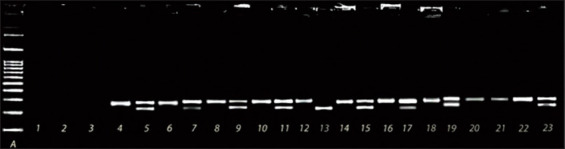
Agarose gel electrophoresis of the polymerase chain reaction (PCR) and restriction fragment length polymorphism (RFLP) products of representative samples tested for the detection of the (SOD1:c.118G>A) gene mutation. Lane A. DNA 100 bp Ladder (Nippon Genetics, Europe GmbH). Lanes 1-3. Negative controls (samples containing water instead of DNA) for DNA isolation (lane 1), PCR (lane 2), and RFLP-PCR (lane 3). Lanes 4, 6, 8, 10, 12, 14, 16, 18, 20, and 22. PCR amplification products of the *SOD1* gene. Lanes 5, 7, 9, 11, 13, 15, 17, 19, 21, and 23. The outcome of RFLP analysis conducted on PCR products using the restriction endonuclease *AcuI* for the GA genotype (lanes 5, 7, 9, 11, 15, 17, 19, and 23), the GG genotype (lane 13), and the AA genotype (lane 21).

An *AcuI* restriction site (CTGAAG(N)_16_↓) is present in the PCR product corresponding to the wild-type *SOD1:c.118G* allele; hence, restriction analysis generates two DNA fragments (230 and 62 bp) in homozygous GG animals. The mutant allele does not present the *Acu1* restriction site; it generates one fragment of 292 bp in homozygous AA animals but three fragments (292, 230, and 62 bp) in heterozygous (GA) animals (Figure-1). The frequency of the G and A alleles was 0.833 and 0.167, respectively.

The observed and expected (under the assumption of HWE) frequencies of the three genotypes are shown in Table-1. The observed relative genotype frequencies were 71% (n=51) for the homozygous (GG), 25% (n=18) for the heterozygous (AG), and 4% (n=3) for the homozygous mutant (AA) alleles. These frequencies were close to those expected for the three genotypes (GG=69%; GA=20%; and AA=3%), indicating no significant departure from HWE (p=0.395). For the locus under study, HWE was also confirmed by the low positive value of the F_IS_ statistic (0.107), which indicated a low heterozygote deficit (18 heterozygous animals observed vs. 20 expected) (Table-1).

**Table-1:** Number of observed and expected genotypes (relative frequencies are reported in parenthesis).

Genotype	Observed	Expected
GG	51 (0.71)	50 (0.69)
GA	18 (0.25)	20 (0.28)
AA	3 (0.04)	2 (0.03)
Total	72 (100)	72 (100)

## Discussion

In the studied group of animals, the frequency of the mutant allele (A) was 0.17, which is slightly higher than that previously reported for Belgian Malinois (0-0.14) [9,18-20] but similar to the frequency reported in other breeds such as the Airedale Terrier, Australian Terrier, Chinese Crested, Chow Chow, French Bulldog, Irish Setter, and Puli [9,18] (Table-2).

**Table-2:** Allele (A) and genotype frequencies of polymorphisms of the SOD1 gene across various canine breeds.

Breed or variety	Genotype frequency			A allele frequency	Reference

	GG	GA	AA		
Affenpinscher	1.00	0.00	0.00	0.00	[9]
Afghan Hound	1.00	0.00	0.00	0.00	[9]
Airedale Terrier	0.50-0.70	0.25-0.30	0.00-0.25	0.15-0.38	[9,18]
Akbash Dog	1.00	0.00	0.00	0.00	[9]
Akita	1.00	0.00	0.00	0.00	[9,18]
Alaskan Husky	1.00	0.00	0.00	0.00	[9,18]
Alaskan Klee Kai	1.00	0.00	0.00	0.00	[9]
Alaskan Malamute	0.98-1.00	0.00	0.00-0.02	0.00-0.02	[9,18]
Alaskan Noble Companion Dog	1.00	0.00	0.00	0.00	[9]
American Bulldog	0.92-1.00	0.00-0.05	0.00-0.03	0.00-0.05	[9,18]
American Eskimo Dog	0.30-0.33	0.35-0.60	0.10-0.33	0.40-0.50	[9,18]
American Foxhound	0.50-1.00	0.00-0.5	0.00	0.00-0.25	[9,18]
American Hairless (Rat) Terrier	0.75	0.25	0.00	0.13	[9]
American Staffordshire Terrier	0.63-1.00	0.00-0.08	0.00-0.29	0.00-0.33	[9,18,^21^]
American Water Spaniel	0.24-0.32	0.45-0.51	0.23-0.25	0.46-0.51	[9,18]
Anatolian Shepherd Dog	0.83	0.13	0.03	0.10	[9]
Argentine Dogo	1.00	0.00	0.00	0.00	[9]
Australian Cattle Dog	0.87-0.89	0.08-0.11	0.00-0.05	0.05-0.09	[9,18]
Australian Kelpie	0.00	0.50	0.50	0.75	[9]
Australian Shepherd	0.00-0.79	0.18-1.00	0.00-0.32	0.12-0.50	[9,18,20]
Australian Terrier	0.50-0.67	0.33-0.50	0.00	0.17-0.25	[9,18]
Basenji	1.00	0.00	0.00	0.00	[9,18]
Basset Hound	1.00	0.00	0.00	0.00	[9,18]
Beagle	0.90-0.91	0.05-0.10	0.00-0.04	0.05-0.06	[9,18]
Bearded Collie	1.00	0.00	0.00	0.00	[9,18]
Beauceron	1.00	0.00	0.00	0.00	[9,18]
Bedlington Terrier	1.00	0.00	0.00	0.00	[9,18]
Belgian Malinois	0.78-1.00	0.00-0.17	0.00-0.06	0.00-0.14	[9,18-20]
Belgian Sheepdog	0.75-0.79	0.11-0.25	0.00-0.11	0.12-0.16	[9,18]
Belgian Tervuren	0.94	0.04	0.02	0.04	[9]
Bergamasco	1.00	0.00	0.00	0.00	[9]
Berger Picard	1.00	0.00	0.00	0.00	[9]
Bernese Mountain Dog	0.37-0.60	0.35-0.46	0.05-0.18	0.23-0.41	[9,18,21]
Bichon Frise	0.96-1.00	0.00	0.00-0.04	0.00-0.04	[9,18]
Black & Tan Coonhound	1.00	0.00	0.00	0.00	[9]
Black Russian Terrier	1.00	0.00	0.00	0.00	[9]
Bloodhound	0.47-0.50	0.44	0.06-0.08	0.28-0.30	[9,18]
Blue Heeler	1.00	0.00	0.00	0.00	[9]
Bluetick Coonhound	0.75	0.00	0.25	0.25	[9]
Boerboel	1.00	0.00	0.00	0.00	[9]
Border Collie	0.79-0.99	0.01-0.09	0.00-0.13	0.008-0.17	[9,18,20-22]
Border Terrier	0.97-1.00	0.00	0.00-0.03	0.00-0.03	[9,18]
Borzoi	0.70-1.00	0.00-0.27	0.00-0.04	0.00-0.17	[9,18]
Boston Terrier	0.93-1.00	0.00	0.00-0.07	0.00-0.07	[9,18]
Bouvier des Flandres	1.00	0.00	0.00	0.00	[9,18]
Boxer	0.12-0.87	0.13-0.34	0.00-0.57	0.07-0.72	[9,18,20,21]
Boykin Spaniel	0.75	0.25	0.00	0.13	[9]
Briard	1.00	0.00	0.00	0.00	[9,18]
Brittany	1.00	0.00	0.00	0.00	[9,18]
Brussels Griffon	1.00	0.00	0.00	0.00	[9]
Bulldog	0.46	0.54	0.00	0.27	[9]
Bullmastiff	0.92	0.00	0.08	0.08	[9]
Bull Terrier	1.00	0.00	0.00	0.00	[9,18]
Cairn Terrier	1.00	0.00	0.00	0.00	[9,18]
Canaan Dog	0.45-0.51	0.40-0.44	0.09-0.11	0.29-0.33	[9,18]
Cane Corso	1.00	0.00	0.00	0.00	[9]
Cardigan Welsh Corgi	0.46-0.50	0.36-0.44	0.10-0.15	0.32	[9,18]
Catahoula Leopard Dog	0.50	0.50	0.00	0.25	[9]
Cavalier King Charles Spaniel	0.09-0.14	0.37-0.82	0.09-0.49	0.50-0.68	[9,18]
Central Asian Sheepdog (Ovcharka)	1.00	0.00	0.00	0.00	[9]
Chesapeake Bay Retriever	0.34-0.41	0.42-0.44	0.15-0.24	0.37-0.45	[9,18]
Chihuahua	1.00	0.00	0.00	0.00	[9]
Chinese Crested	0.63-0.75	0.25-0.37	0.00	0.12-0.18	[9,18]
Chinese Shar Pei	1.00	0.00	0.00	0.00	[9,18]
Chinook	1.00	0.00	0.00	0.00	[9,18]
Chow Chow	0.64-0.70	0.27-0.32	0.03-0.05	0.16-0.20	[9,18]
Clumber Spaniel	0.91	0.09	0.00	0.05	[9,18]
Cocker Spaniel (American)	0.97-1.00	0.00-0.03	0.00	0.00-0.01	[9,18]
Collie	0.48-0.76	0.24-0.28	0.00-0.26	0.12-0.39	[9,18,23]
Coton de Tulear	0.85-0.87	0.13-0.15	0.00	0.07	[9,18]
Curly Coated Retriever	1.00	0.00	0.00	0.00	[9,18]
Czechoslovakian Wolfdog	0.46-0.54	0.40-0.43	0.04-0.13	0.25-0.34	[9,24]
Dachshund	1.00	0.00	0.00	0.00	[9,18]
Dalmatian	0.96-0.97	0.02-0.03	0.00-0.02	0.03-0.15	[9,18]
Dandie Dinmont Terrier	1.00	0.00	0.00	0.00	[9,18]
Decker Terrier	0.88	0.13	0.00	0.06	[9]
Doberman Pinscher	0.98-1.00	0.00	0.00-0.02	0.00-0.02	[9,18]
Dogue du Bordeaux	1.00	0.00	0.00	0.00	[9,18]
Dutch Shepherd	0.00-0.83	0.17-1.00	0.00	0.08-0.50	[9,18]
English Bulldog	0.83	0.17	0.00	0.08	[20]
English Cocker Spaniel	1.00	0.00	0.00	0.00	[9,18]
English Coonhound	0.88	0.13	0.00	0.06	[9]
English Foxhound	1.00	0.00	0.00	0.00	[9,18]
English Setter	1.00	0.00	0.00	0.00	[9,18]
English Shepherd	0.89-0.90	0.10-0.11	0.00	0.05-0.06	[9,18]
English Springer Spaniel	0.76-0.78	0.13-0.19	0.03-0.10	0.12-0.17	[9,18]
English Toy Spaniel	0.59	0.38	0.03	0.22	[9]
Field Spaniel	1.00	0.00	0.00	0.00	[9,18]
Finnish Lapphund	0.87	0.13	0.00	0.06	[9,18]
Finnish Spitz	0.97	0.03	0.00	0.02	[9,18]
Flat-Coated Retriever	0.92-1.00	0.00-0.05	0.00-0.03	0.00-0.05	[9,18]
Fox Terrier-Smooth	1.00	0.00	0.00	0.00	[9,18]
Fox Terrier-Wire	0.01-0.03	0.09-0.15	0.82-0.90	0.90-0.94	[9,18]
French Bulldog	064-0.74	0.21-0.27	0.06-0.09	0.16-0.23	[9,18]
German Pinscher	0.93	0.07	0.00	0.03	[9,18]
German Shepherd Dog	0.44-0.81	0.13-0.37	0.03-0.22	0.13-0.38	[7-9,18-21]
German Shorthaired Pointer	0.91-1.00	0.00-0.02	0.00-0.07	0.00-0.08	[9,18]
German Wirehaired Pointer	0.91	0.09	0.00	0.05	[9]
Giant Schnauzer	0.98-1.00	0.00	0.00-0.02	0.00-0.02	[9,18]
Glen of Imaal Terrier	1.00	0.00	0.00	0.00	[9]
Golden Retriever	0.96-1.00	0.00-0.01	0.00-0.03	0.00-0.03	[9,18,21]
Gordon Setter	0.98-1.00	0.00	0.00-0.03	0.00-0.03	[9,18]
Great Dane	1.00	0.00	0.00	0.00	[9,18]
Great Pyrenees	0.80-0.81	0.12-0.15	0.04-0.08	0.12-0.14	[9,18]
Greater Swiss Mountain Dog	1.00	0.00	0.00	0.00	[9,18]
Greyhound	0.97	0.00	0.03	0.03	[9]
Hanoverian Hound	1.00	0.00	0.00	0.00	[9]
Harrier	0.81-0.86	0.14-0.19	0.00	0.07-0.10	[9,18]
Havana Silk Dog	1.00	0.00	0.00	0.00	[9]
Havanese	1.00	0.00	0.00	0.00	[9,18]
Hovawart	0.48	0.27	0.25	0.38	[9]
Ibizan Hound	1.00	0.00	0.00	0.00	[9,18]
Icelandic Sheepdog	1.00	0.00	0.00	0.00	[9,18]
Irish Red and White Setter	1.00	0.00	0.00	0.00	[9,18]
Irish Setter	0.68-0.74	0.21-0.29	0.03-0.05	0.16-0.18	[9,18]
Irish Terrier	0.90	0.10	0.00	0.05	[9,18]
Irish Water Spaniel	1.00	0.00	0.00	0.00	[9,18]
Irish Wolfhound	0.93-0.94	0.06-0.07	0.00	0.03	[9,18]
Italian Greyhound	1.00	0.00	0.00	0.00	[9,18]
Jack Russell Terrier	0.60-0.78	0.17-0.19	0.04-0.23	0.13-0.32	[9,18]
Japanese Chin	1.00	0.00	0.00	0.00	[9,18]
Karelian Bear Dog	1.00	0.00	0.00	0.00	[9]
Keeshond	0.97-0.98	0.02-0.03	0.00	0.01-0.02	[9,18]
Kerry Blue Terrier	0.46	0.35-0.40	0.15-0.19	0.34-0.37	[9,18]
King Shepherd	0.56	0.28	0.17	0.31	[9]
Komondor	0.58	0.40	0.02	0.22	[9]
Kuvasz	0.61-0.65	0.25-0.27	0.08-0.13	0.22-0.26	[9,18]
Labrador Retriever	0.91-1.00	0.00-0.04	0.00-0.05	0.00-0.07	[9,18,21]
Leonberger	1.00	0.00	0.00	0.00	[9,18]
Lhasa Apso	1.00	0.00	0.00	0.00	[9,18]
Lowchen	1.00	0.00	0.00	0.00	[9,18]
Maltese	1.00	0.00	0.00	0.00	[9]
Manchester Terrier-Standard	1.00	0.00	0.00	0.00	[9,18]
Manchester Terrier-Toy	1.00	0.00	0.00	0.00	[9,18]
Maremma	0.50	0.00	0.50	0.50	[9]
Mastiff (English Mastiff)	0.77-0.82	0.17-0.19	0.02-0.03	0.10-0.13	[9,18]
Miniature Bull Terrier	1.00	0.00	0.00	0.00	[9,18]
Miniature Pinscher	1.00	0.00	0.00	0.00	[9]
Miniature Schnauzer	0.96-1.00	0.00	0.00-0.04	0.00-0.04	[9,18]
Mountain Cur	0.00	0.00	1.00	1.00	[9]
Mudi	1.00	0.00	0.00	0.00	[9]
Native Am Indian Dog	1.00	0.00	0.00	0.00	[9]
Neapolitan Mastiff	1.00	0.00	0.00	0.00	[9,18]
Newfoundland	0.98-1.00	0.00	0.00-0.02	0.00-0.02	[9,18]
Norwegian Buhund	1.00	0.00	0.00	0.00	[9]
Norwegian Elkhound	1.00	0.00	0.00	0.00	[9]
Norwegian Lundehund	1.00	0.00	0.00	0.00	[9,18]
Norfolk Terrier	0.00	0.67	0.33	0.67	[9]
Norwich Terrier	0.81-1.00	0.00-0.19	0.00	0.00-0.09	[9,18]
Nova Scotia Duck Tolling Retriever	0.78-0.88	0.10-0.19	0.02-0.03	0.07-0.13	[9,18]
Olde English Bulldogge	0.50	0.25	0.25	0.38	[9]
Old English Sheepdog	0.89-1.00	0.00-0.11	0.00	0.00-0.06	[9,18,20]
Otterhound	1.00	0.00	0.00	0.00	[9,18]
Papillon	1.00	0.00	0.00	0.00	[9,18]
Parson Russell Terrier	1.00	0.00	0.00	0.00	[9,18]
Patterdale Terrier	0.00	0.00	1.00	1.00	[9]
Pembroke Welsh Corgi	0.00-0.09	0.28-0.43	0.48-0.68	0.697-0.83	[6,9,18,20]
Perro de Presa Canario	1.00	0.00	0.00	0.00	[9]
Peruvian Inca Orchid	0.00	0.00	1.00	1.00	[9]
Petit Basset Griffon Vendeen	1.00	0.00	0.00	0.00	[9,18]
Pharaoh Hound	0.97-1.00	0.00	0.00-0.03	0.00-0.03	[9,18]
Pit Bull Terrier	0.43	0.11	0.45	0.51	[9]
Plott	0.96	0.04	0.00	0.02	[9]
Pointer	0.92-1.00	0.00	0.00-0.08	0.00-0.08	[9,18]
Polish Lowland Sheepdog	1.00	0.00	0.00	0.00	[9]
Pomeranian	0.76-0.79	0.18-0.21	0.03	0.12-0.14	[9,18]
Poodle-Miniature	0.93	0.07	0.00	0.04	[9]
Poodle-Standard	0.88-0.91	0.06-0.12	0.00-0.03	0.06-0.07	[9,18,20]
Poodle-Toy	0.75	0.25	0.00	0.13	[9]
Portuguese Podengo	0.97	0.03	0.00	0.02	[9]
Portuguese Pointer	1.00	0.00	0.00	0.00	[9]
Portuguese Water Dog	1.00	0.00	0.00	0.00	[9,18]
Pug	0.46-0.67	0.30-0.33	0.00-0.23	0.17-0.38	[9,18,^20^]
Puli	0.71	0.24	0.06	0.17	[9]
Pumi	0.88	0.13	0.00	0.06	[9]
Pyrenean Shepherd	1.00	0.00	0.00	0.00	[9,18]
Rat Terrier	0.98	0.02	0.00	0.01	[9]
Redbone Coonhound	1.00	0.00	0.00	0.00	[9]
Rhodesian Ridgeback	0.46-0.52	0.39-0.42	0.09-0.11	0.28-0.33	[9,18]
Romanian Mioritic Shepherd	1.00	0.00	0.00	0.00	[9]
Rottweiler	0.86-0.96	0.03-0.14	0.00-0.01	0.02-0.07	[9,18,20]
Russell Terrier	0.75	0.25	0.00	0.13	[9]
Saint Bernard	0.77-1.00	0.00-0.19	0.00-0.04	0.00-0.13	[9,18]
Saluki	0.92-1.00	0.00-0.04	0.00-0.04	0.00-0.06	[9,18]
Samoyed	0.98-1.00	0.00-0.02	0.00	0.00-0.01	[9,18]
Sapsaree	1.00	0.00	0.00	0.00	[9]
Schipperke	1.00	0.00	0.00	0.00	[9,18]
Scottish Deerhound	1.00	0.00	0.00	0.00	[9,18]
Scottish Terrier	1.00	0.00	0.00	0.00	[9,18]
Sealyham Terrier	0.70-0.71	0.29-0.30	0.00	0.15	[9,18]
Shetland Sheepdog	0.71-0.83	0.17	0.00-0.12	0.09-0.21	[9,18]
Shiba Inu	1.00	0.00	0.00	0.00	[9,18]
Shih Tzu	0.38-0.43	0.29-0.50	0.13-0.29	0.09-0.43	[9,18]
Shiloh Shepherd	0.73	0.24	0.03	0.15	[9]
Siberian Husky	0.95-1.00	0.00-0.03	0.00-0.02	0.00-0.04	[9,18]
Siberian Laika	0.00	1.00	0.00	0.50	[9]
Silken Windhound	1.00	0.00	0.00	0.00	[9]
Silky Terrier	0.74	0.16	0.10	0.18	[9]
Skye Terrier	1.00	0.00	0.00	0.00	[9]
Small Munsterlander	1.00	0.00	0.00	0.00	[9]
Soft Coated Wheaten Terrier	0.51-0.83	0.17-0.20	0.00-0.28	0.09-0.39	[9,18]
Spinone Italiano	1.00	0.00	0.00	0.00	[9,18]
Staffordshire Bull Terrier	0.88-0.94	0.00-0.02	0.06-0.10	0.06-0.11	[9,18]
Standard Schnauzer	0.97-1.00	0.00-0.03	0.00	0.00-0.01	[9,18]
Sussex Spaniel	1.00	0.00	0.00	0.00	[9,18]
Swedish Valhund	1.00	0.00	0.00	0.00	[9,18]
Tamaskan (Aatu Tamaskan)	0.68	0.29	0.03	0.18	[9]
Tenterfield Terrier	0.89	0.11	0.00	0.05	[9]
Tibetan Mastiff	1.00	0.00	0.00	0.00	[9,18]
Tibetan Spaniel	1.00	0.00	0.00	0.00	[9,18]
Tibetan Terrier	0.46-0.53	0.35	0.12-0.19	0.29-0.36	[9,18]
Toy Fox Terrier	1.00	0.00	0.00	0.00	[9,18]
Treeing Walker Coonhound	0.95	0.00	0.05	0.05	[9]
Vizsla	1.00	0.00	0.00	0.00	[9,18]
Volpino Italiano	1.00	0.00	0.00	0.00	[9]
Wachtelhund (German Spaniel)	0.00	1.00	0.00	0.50	[9]
Weimaraner	1.00	0.00	0.00	0.00	[9,18]
Welsh Springer Spaniel	1.00	0.00	0.00	0.00	[9,18]
Welsh Terrier	0.56-0.57	0.35	0.08-0.10	0.26-0.27	[9,18]
West Highland White Terrier	1.00	0.00	0.00	0.00	[9,18]
Whippet	1.00	0.00	0.00	0.00	[9,18]
Wirehaired Pointing Griffon	1.00	0.00	0.00	0.00	[9,18]
Wire Fox Terrier	0.01	0.09	0.90	0.94	[9]
Yorkshire Terrier	0.75	0.25	0.00	0.13	[9]
hybrid/mix-breed	0.41-0.44	0.11-0.16	0.39-0.48	0.48-0..53	[9,18]

The heterozygosity and homozygosity rates observed were similar to those previously recorded in the same breed (0.25 vs. 0–0.17 and 0.04 vs. 0–0.06 for heterozygosity and homozygosity, respectively) [9,18-20] and other breeds (Airedale Terrier, American Hairless Terrier, Beagle, Belgian Sheepdog, Borzoi, Boxer, Boykin, Chow Chow, Spaniel, and Kuvasz) (Table-2) [6-9,18-24].

The appreciable frequency of heterozygous (carriers) animals indicates that selective breeding is necessary to reduce the high risk of developing CDM in forthcoming generations of this Belgian Malinois population: Mating among carriers would result in 25% AA progeny. However, genetic selection using the *SOD1:c.118G>A* mutation against CDM must be carefully conducted to avoiding genetic erosion caused by inbreeding and the reduction of the effective population size. Notably, the management practice applied to the studied group of animals by the Hellenic Armed Forces aimed to avoid inbreeding, mainly by selecting individuals from different breeders in Greece based on breed standards and pedigree records.

Medical records were analyzed, and it was noteworthy that the targeted population had been maintained under a “test and remove” practice against leishmaniosis; this practice had been applied across generations by breeders and the Hellenic Army. The fact that the tested population was under HWE may be associated with the impact of this selection criterion on the genetic constitution of the population, particularly the frequency of the mutant allele [25]. This is consistent with the role of the wild-type *SOD1* gene in building a strong immune response against intracellular pathogens such as *Leishmania* spp. [26-29]. Thus, the removal of *Leishmania*-positive individuals from the parental population of the tested animals for several generations may have decreased the frequency of the mutant allele to the level observed in this study. Notably, the link between DM and infectious myelopathy, a typical feature of canine leishmaniosis, is also supported by the association of pathogenesis in CDM-analogous diseases of cats and humans, that is, feline DM and ALS, with the feline leukemia virus [30-32] and retrovirus infections, respectively [11,13].

## Conclusion

The *SOD1:c.118G>A* mutation was common in the tested group of dogs; therefore, this group is suitable for a follow-up assessment of the development and progression of CDM. A case-control study on animals with evidence of sensitivity to infectious myelopathy could also provide new insights into disease pathogenesis.

## Authors’ Contributions

AM: Conducted research in the laboratory with support from GSZ and ALH. JI: Designed the research and drafted the manuscript with support from AK and AM. CDV: Collected the samples for this study. AK: Collected data and conducted genetic analysis. AM, JI, NT, and MG: Reviewed and updated the manuscript. All authors read and approved the final manuscript.
